# Exercise-Induced Hypoalgesia in Patients with Chronic Whiplash-Associated Disorders: Differences between Subgroups Based on the Central Sensitization Inventory

**DOI:** 10.3390/jcm13020482

**Published:** 2024-01-15

**Authors:** Erwin Hendriks, Iris Coppieters, Lennard Voogt, Wilfried Cools, Kelly Ickmans

**Affiliations:** 1 Pain in Motion Research Group, Department of Physiotherapy, Human Physiology and Anatomy, Faculty of Physical Education & Physiotherapy, Vrije Universiteit Brussel, Laarbeeklaan 103, 1090 Brussels, Belgium; erwin.paul.hendriks@vub.be (E.H.); iris.coppieters@vub.be (I.C.); l.p.voogt@hr.nl (L.V.); 2 Rehabilitation Centre Drechtsteden/Haaglanden, Berkenhof 100, 3319 WB Dordrecht, The Netherlands; 3 Research Centre for Health Care Innovations, Rotterdam University of Applied Sciences, Rochussenstraat 198, 3015 EK Rotterdam, The Netherlands; 4 Unit Physiotherapy, Organizational Part of the Orthopedics Department, Erasmus Medical Centre, Doctor Molewaterplein 40, 3015 GD Rotterdam, The Netherlands; 5 Laboratory for Brain-Gut Axis Studies (LaBGAS), Translation Research in Gastrointestinal Disorders (TARGID), Department of Chronic Diseases and Metabolism (CHROMETA), KU Leuven, Oude Markt 13, 3000 Leuven, Belgium; 6 Core Facility—Support for Quantitative and Qualitative Research (SQUASH), Vrije Universiteit Brussel, Pleinlaan 2, 1050 Brussels, Belgium; wilfried.cools@vub.be; 7 Department of Physical Medicine and Physiotherapy, Universitair Ziekenhuis Brussel, Laarbeeklaan 101, 1090 Brussels, Belgium; 8 Movement & Nutrition for Health & Performance Research Group (MOVE), Department of Movement and Sport Sciences, Faculty of Physical Education and Physiotherapy, Vrije Universiteit Brussel, Pleinlaan 2, 1050 Brussels, Belgium

**Keywords:** central sensitization, chronic whiplash-associated disorder, exercise-induced hypoalgesia, central sensitization inventory, central sensitization inventory symptom severity calculator, subgroups

## Abstract

Background: Physical exercise is an important element in the rehabilitation of chronic whiplash-associated disorders, with the physiological process underlying pain reduction called exercise-induced hypoalgesia. In chronic whiplash-associated disorders, exercise-induced hypoalgesia appears impaired, and the research suggests a relationship with symptoms of dysfunctional nociceptive processing, such as central sensitization. This study improves our understanding of exercise-induced hypoalgesia in chronic whiplash-associated disorders by examining the differences between the extent of exercise-induced hypoalgesia in subgroups based on scores on the central sensitization inventory (CSI). Methods: Data were collected from 135 participants with chronic whiplash-associated disorders who completed a set of questionnaires. Pain pressure thresholds and temporal summations were assessed before and after a submaximal aerobic bicycle exercise test. Results: We observed no interaction effect between exercise-induced hypoalgesia and the CSI scores for both pain pressure threshold and temporal summation. No overall statistical effect was measured in the analysis of the effect of time. The pain pressure threshold significantly related to the CSI. The temporal summation showed no correlation. Conclusions: During this study, we did not find evidence for a difference in the presence of exercise-induced hypoalgesia when the subgroups were created based on the central sensitization cluster calculator. Limited evidence was found for the influence of CSI scores on the delta pain pressure threshold.

## 1. Introduction

Persistent pain is common in chronic whiplash-associated disorders (WADs) [1,2,3,4,5,6]. The research indicates that performing exercise is beneficial for patients with various chronic pain conditions and positively influences pain severity, overall physical and mental health, and, therefore, the quality of life [6,7,8]. Although strict guidelines for conducting physical activity when experiencing chronic pain are lacking, physical exercise is a cornerstone in rehabilitation programs for patients with chronic pain, including WAD [8,9]. Engaging in different types of exercise have been found to reduce pain sensitivity, a phenomenon known as ‘exercise-induced hypoalgesia’ [10,11,12]. This effect entails a decrease in sensitivity to both non-painful and painful stimuli, lasting up to 30 min following a single bout of exercise [8]. The mechanisms explaining exercise-induced hypoalgesia are still not fully understood, but the research consistently shows reductions in pain sensitivity after a bout of exercise, suggesting that central or systemic mechanisms are involved [12]. It is suggested that exercise-induced hypoalgesia can occur through an increase in beta-endorphins, altered psychological states, the interaction between cardiovascular and pain processing systems, recruitment of high-threshold motor units, and activation of the primary motor cortex [12]. Hypotheses explaining exercise-induced hypoalgesia are variable in this regard, and a possible factor is conditioned pain modulation, being an interesting aspect to explore [8,12,13,14,15,16]. Conditioned pain modulation is a psychophysical experimental measure of endogenous pain inhibitory pathways, where pain from a noxious stimulus activates descending inhibitory pathways. This activation leads to a decrease in pain during a second noxious stimulus applied elsewhere [12,17]. Conditioned pain modulation can be tested by quantitative sensory testing (QST) [18]. Quantitative sensory testing comprises a set of procedures that assess perceptual responses to systematically applied and quantifiable sensory stimuli. Its goal is to characterize somatosensory function or dysfunction and to assess the integrity of the entire neural axis from receptor to brain [19,20]. Various stimuli are commonly used to assess experimental pain responses, but thermal and mechanical stimuli are typically employed [19].

Different studies have demonstrated exercise-induced hypoalgesia following aerobic and isometric exercises in healthy, pain-free individuals [11,21,22]. The response in chronic pain populations is more variable with pain sensitivity decreasing, remaining unchanged, or, in some cases, even increasing in response to exercise [9]. Patients with chronic WADs may also present with dysfunctional pain inhibition and there are inconclusive results regarding what is the most appropriate form of exercise, for example, aerobic versus isometric exercises, to reduce pain sensitivity in people with chronic WADs [1]. The research reveals impaired exercise-induced hypoalgesia in individuals with chronic WADs both at rest and after performing submaximal aerobic exercises [23,24,25,26,27]. The reasons are likely multifactorial, but the findings suggest impaired exercise-induced hypoalgesia occurs more frequently in individuals with dysfunctional central nociceptive processing, also called central sensitization [8,28,29]. Central sensitization comprises generalized hypersensitivity to various stimuli caused by the amplification of neural signaling in the central nervous system [30]. The presence of central sensitization in chronic WAD has been established [31,32]. Hypothetically, there might be a relationship between the variation of exercise-induced hypoalgesia and the extent of central sensitization-related symptoms present in individuals with chronic WADs. At present, it is, however, unknown to what extent there is a relationship between exercise-induced hypoalgesia and different degrees of central sensitization-related symptoms. Further research of exercise-induced hypoalgesia in people with chronic WADs is warranted.

The central sensitization inventory (CSI) was developed to measure somatic and emotional symptoms commonly observed in individuals with central sensitization. It is widely utilized in scientific research and clinical practice [33]. A study by Cuesta-Vargas and colleagues provided a CSI symptom severity calculator that made it possible to classify patients into three cluster groups labeled (i) low level, (ii) medium level, and (iii) high level of central sensitization-related symptom severity [34]. The purpose of this study is to assess whether there is a difference in the level of exercise-induced hypoalgesia in people with chronic WADs when subgroups are formed based on central sensitization-related symptoms. We hypothesize that the extent of exercise-induced hypoalgesia is lower when more central sensitization-related symptoms are present.

## 2. Materials and Methods

### 2.1. Study Design and Settings

A cross-sectional study was conducted, in line with the STROBE Statement (www.strobe-statement.org/ (accessed on 12 June 2016)), investigating the effect of the CSI score on exercise-induced hypoalgesia in a group of participants with chronic WADs.

The study was conducted at the Rehabilitation Centre Drechtsteden and LENGG Rehabilitation in Dordrecht, the Netherlands. The participants were recruited from patients referred for multidisciplinary chronic pain management rehabilitation, and patients consecutively presenting to the clinic with a whiplash injury were approached. Eligible participants were informed by telephone, in person, by letter, or by e-mail about the study. Participants willing to participate signed an informed consent form before enrolling in the study. The medical ethics committee of the Maasstad Hospital Rotterdam, The Netherlands, approved the study.

### 2.2. Participants

The inclusion criteria for participants were as follows: Dutch speaking, aged between 18 to 65 years, having experienced a whiplash trauma at least 3 months ago, and having experienced a mean pain score of 4 or more on a numeric rating scale of 10 over the last month, aligning with the criteria used in similar studies [1,26]. People were excluded if they fulfilled the criteria of a grade IV WAD injury defined by the Quebec Task Force classification (i.e., fracture or dislocation of the cervical spine) [35], were diagnosed with other chronic diseases (e.g., fibromyalgia, rheumatologic diseases, neurological diseases, psychiatric diseases, cardiovascular diseases, or diabetes mellitus), and had a history of neck or shoulder surgeries. As it is known that women with pre-existing chronic pain states can experience an attenuation of symptoms during and after pregnancy [36,37,38,39], the participants were also excluded if they were pregnant or had given birth in the last year before the study enrolment period.

### 2.3. Procedure

A test protocol was developed and implemented. The participants initiated the process by completing a set of questionnaires as the baseline measurements. The questionnaires were filled out on paper without the supervision of a researcher. After the collection of the baseline measurements, a submaximal aerobic bicycle exercise was performed, combined with quantitative sensory testing procedures before and after the aerobic exercises. This protocol has previously been used to document the lack and/or the presence of exercise-induced hypoalgesia in populations of people with chronic fatigue syndrome and patients with chronic pain resulting from chronic WADs, rheumatoid arthritis, knee osteoarthritis, and/or fibromyalgia [24,25,29,40,41,42]. If a participant could not complete the questionnaires prior to aerobic exercise, for example, due to an increase in physical complaints, we continued with the rest of the test protocol as planned. In such cases, the questionnaires were completed after the aerobic exercise, taken home and sent back by regular post, or brought back at the beginning of the rehabilitation program. Participants were instructed to complete the questionnaires as soon as possible, but at least in the same week.

### 2.4. Measurements

#### 2.4.1. Baseline Questionnaires

The participants filled out the RAND-36 item health survey (RAND-36), neck disability index (NDI), the modified perceived deficits questionnaire (mPDQ), and their mean pain experience in the last week on a visual analog scale (VAS) for descriptive purposes. The RAND-36 is a validated tool for health-related quality of life, and the Dutch version is approved as both valid and reliable [43,44,45]. Higher scores indicated a better health-related quality of life. The Dutch version of the NDI is a valid instrument of self-reported neck pain disability with good reliability and responsiveness, where higher scores represent more disabilities [46,47,48,49]. The Dutch version of the mPDQ is a valid and reliable questionnaire used to understand perceived cognitive problems with high internal consistencies [50,51]. The VAS is the most widely used tool for assessing pain and is a valid and reliable method for evaluating pain, where higher scores indicate a greater experience of pain [52,53].

#### 2.4.2. Central Sensitization Inventory (CSI)

The CSI has been validated in multiple languages, and the Dutch version demonstrates good psychometric properties [54,55]. It consists of 2 parts, of which part A includes 25 items about central sensitization-related symptoms, scored on a 5-point Likert scale ranging from 0 to 4 [56]. Higher total scores indicate a higher degree of central sensitization symptomology. Part B evaluates previously diagnosed central sensitization-related disorders, which this study does not consider for its analysis. Previous research provided CSI severity levels as a guideline for interpreting CSI scores: subclinical = 0 to 29; mild = 30 to 39; moderate 40 to 49; severe 50 to 59; and extreme = 60 to 100 [57]. Additionally, a cut-off score of 40 provided a clinically relevant threshold, alerting healthcare professionals to the possibility that a patient’s symptom presentation could indicate the presence of central sensitization syndromes [33,58].

#### 2.4.3. Submaximal Aerobic Bicycle Exercise Test

The standardized submaximal bicycle ergometer test used in this study is known as the ‘aerobic power index’ test, representing the aerobic component of a series of three tests comprising the tri-level fitness profile developed by Telford and colleagues [59]. The test has been shown to be reliable in healthy people and a number of clinical populations, including patients with chronic fatigue syndrome, cancer, and people with a sedentary lifestyle and/or are obese [60,61,62,63]. This test has been previously used in the research on populations with chronic WADs [23,25,26]. Prior to starting the test, the target heart rate was calculated as 75% of the age-predicted maximum heart rate (0.75 × (220—age in years). The aerobic power index test has a low starting point of 25 watt that increases by 25 watt every minute until the individual target heart rate is reached [59]. If the participants were unable to reach their individual target heart rates, they were motivated to continue the test for as long as possible. This approach was hypothesized to result in effective submaximal exercise, as was the goal of the aerobic power index test.

#### 2.4.4. Quantitative Sensory Testing (QST)

We collected pain pressure thresholds (PPTs) and temporal summation (TS) measurements during this study. Measurements were performed unilaterally on the right side of the body, the upper trapezius muscle (midway between C7 and the lateral border of the acromion), and the quadriceps (in the middle between the anterior superior iliac spine and upper patella border). This procedure has been described before in quantitative sensory testing measurements for people with chronic WADs [25,64,65]. Pressure was applied with a consistent rate of 1 kg/cm^2^/s using a digital algometer equipped with a 1 cm^2^ tip (Wagner Instruments, Greenwich, CT, USA) [66]. The pain pressure threshold was defined as the minimum pressure in kilograms at which the participant first reported experiencing an unpleasant sensation, consistent with similar research [25,31,67,68,69]. Pain pressure thresholds were determined twice at each test side (30 s apart), and the definitive pain pressure threshold was calculated as the mean of these 2 measurements in units of kilogram-force (kg/cm^2^). The stimulus conditions followed those applied in previous studies [27,64,70]. Temporal summation was assessed by the application of a series of short noxious stimuli, believed to represent the wind-up physiological phenomenon and interpreted as the gradual increase in pain sensitivity when continuously exposed to stimuli with a constant intensity [71,72]. A train of ten pulses was administered at the previously determined mean pain pressure threshold intensity on the upper trapezius muscle and the quadriceps at a rate of 1 pulse per second. Temporal summation was examined 2 min after the final pain pressure threshold had been measured to ensure the temporal summation pulses were not influenced by possible sensitization from any previous pressure stimulation. The participants rated the painfulness of the first, fifth, and tenth stimuli on a verbal numeric rating scale (VNRS) ranging from 0 (=no pain) to 10 (=worst possible pain). The outcome measure for the temporal summation was the difference between the tenth and the first VNRS scores, as described by Cathcart et al. [66].

#### 2.4.5. Exercise-Induced Hypoalgesia

Endogenous pain modulation is a wide-ranging term, delineating the array of actions that the central nervous system can use to reduce or increase the experience of pain [71]. In this study, we investigated the effectiveness of endogenous pain inhibition by exploring exercise-induced hypoalgesia [73,74]. Exercise-induced hypoalgesia was assessed through quantitative sensory testing, conducted both at the affected area and a segmentally unrelated area of the body. We assessed exercise-induced hypoalgesia by calculating the difference of the mean pain pressure threshold and the temporal summations before and after the aerobic power index test. This procedure has been previously utilized for the assessment of exercise-induced hypoalgesia [25,75]. Pain pressure thresholds and temporal summations were collected immediately following the aerobic exercise. We interpreted exercise-induced hypoalgesia to have occurred where a significant positive difference was calculated between the pain pressure threshold and temporal summation post-exercise in comparison to pre-exercise. This was when the pain pressure threshold and temporal summation were greater post-exercise than pre-exercise.

### 2.5. Statistical Analyses

We performed a linear mixed-effects analysis to examine the influence of the CSI score on the pain pressure threshold and temporal summation scores (QST measurements) before and after the aerobic power index tests, while accounting for the repeated-measurements structure of the data [76]. We focused on assessing the role of the CSI in predicting pain pressure threshold and temporal summation scores in response to the exercise intervention. Therefore, we examined the differences in the quantitative sensory testing measurements between conditions with lower and higher CSI scores following the responses to exercise intervention. During this study, we used Akaike information criterion (AIC) model selection for the statistical analysis. The overall fit of the regression model to the observed data was measured. The model included “log PPT and TS” (PPT and TS scores), “region” (trapezius and quadriceps), “time” (pre- and post-aerobic exercises), “CSI” (CSI score), and “log-PPT/TS~CSI + time × region” as the fixed effects, along with a random intercept for each participant. *p*-values were obtained by likelihood ratio tests of the entire model with the effect in question against the model without the effect in question. The significance level was set at *p* < 0.05. Given the skewed data, a log-transformation was applied, and, subsequently, the boxplot for the logarithmic transformation of the pain pressure threshold showed this to be a solution. For the temporal summation data, which was not skewed and lacked exceptionally high residuals, no transformation was necessary. The normality of the data was assessed using the Shapiro–Wilk test, and visual inspections were performed through histograms and boxplots [77,78]. The visual inspection of residuals plots revealed an obvious deviation from normality for the pain pressure threshold measurements. There were high residuals in the pain pressure threshold score, but removing this did not alter the outcome of the statistics. Consequently, outliers were not removed. We used R for the statistical analysis.

## 3. Results

During August 2016 and July 2020, the data collection took place and 184 patients with chronic WADs were referred for treatment. After screening for exclusion criteria and an assessment of the possibility for participating in the study, 34 participants were excluded, and a total of 150 patients was eligible to enroll in the study. Of those, 140 patients decided to participate after receiving invitations. The 10 participants who declined did so because of the expectation that it would be too burdensome to participate (*n* = 6) or had no interest in participating in the test *(n* = 4). From 4 participants who joined the study, no data were received or completed outside the specified timeframe of the test protocol. When the measurements were completed, one participant turned out to be pregnant unknowing of that situation at the time of the tests, so that person was excluded later on from the study as well. Ultimately, the data of a total of 135 participants were used for the analysis. The demographics of the participants are presented in Table 1.

From the 184 patients referred to the rehabilitation center during the data collection period, 31 participants were excluded because of the following reasons: insufficient ability to speak Dutch (*n* = 1), aged below 18 years (*n* = 2), experienced whiplash trauma less than 3 months ago (*n* = 1), experienced a mean pain score of less than 4 on a numeric rating scale of 10 over the last month (*n* = 3), diagnosed with other chronic diseases (*n* = 18), had a history of neck or shoulder surgeries (*n* = 2), were pregnant or gave birth in the last year prior to the study enrolment period (*n* = 1), and presented a combination of different exclusion criteria (*n* = 3). In addition, the possibility of eligibility for the study was erroneous and a participant was incorrectly not included (*n* = 1). Due to COVID-19 regulations, measurements could not be conducted on 2 participants (*n* = 2), and despite fulfilling the inclusion criteria, they were unable to participate in the study.

Not all participants completed the study, resulting in missing data and a discrepancy in participant numbers (*n*) in different tables. For each measurement, we specified the number of participants from whom we were able to collect data. This was pointed out by the number, ‘*n*’, mentioned in the table. Missing data resulted from participants finding the measurements too stressful to complete, but who were willing to participate in a part of the test protocol. The multilevel analysis allowed us to counteract the loss of data due to dropouts.

### 3.1. Self-Reported Perceived Complaints

The measurements of the study population are presented in Table 2. The reported RAND-36 scores are comparable with other research populations of chronic WAD, though our population exhibits lower scores in the subscales of physical and social functioning roles [79,80,81,82,83]. The NDI score is comparable with the described NDI scores of chronic WAD patients in some previous studies [84,85,86], although other studies reported lower outcomes for the NDI scores in a cohort of patients with WADs [23,26]. The mean mPDQ was substantially higher than that reported in other studies using cohorts of women [50,68]. The mean VAS score in the last week was higher than that reported in other studies with chronic WAD [23,26].

The CSI cluster calculator was utilized to form three subgroups based on the severity of central sensitization symptoms [34]. The results show an uneven distribution across the different subgroups. In total, 4 participants (3.1%) were classified as having a low level (mild cluster), 11 participants (8.7%) were classified as having a medium level (moderate cluster), and 112 participants (88.2%) were classified as having a high level (severe cluster) of central sensitization-related symptom severity.

### 3.2. Quantitative Sensory Testing

The results of the quantitative sensory testing before and after aerobic exercises for patients with chronic WADs were compared with the influence of the CSI score. The outcomes of the differences in the pain pressure threshold (ΔPPT) and temporal summation (ΔTS) before and after aerobic exercises are displayed in Table 3.

We fitted a linear mixed model (estimated using the restricted maximum likelihood) to predict the log pain pressure threshold and temporal summation scores with the CSI and time (formula = log PPT + 1 ~ CSI + time × region). The model included regionT and residual as random effects (formula = ~1 + region|id). Standardized parameters were obtained by fitting the model on a standardized version of the dataset. Region was included as a random parameter to allow individuals to differ in the effects of the region, with some showing more differences than others. Because the region explained the difference in a side analysis, it was introduced as an interaction effect.

**Figure 1:**
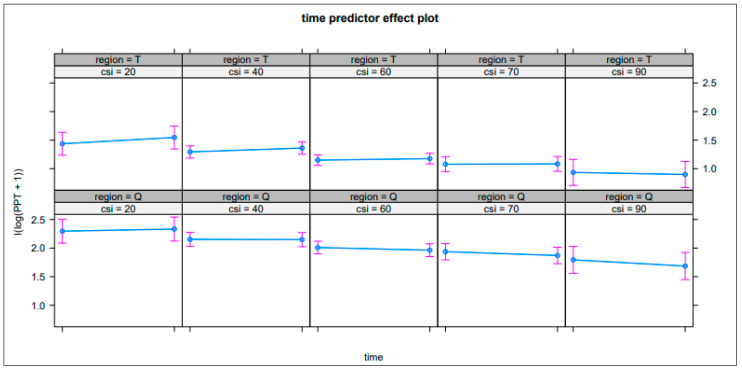
Interaction by CSI score and region. Region = T—region trapezius, region = Q—quadriceps region, CSI—score for the central sensitization inventory, log-PPT—the distribution of the PPT score.

The model’s intercept value, corresponding to log PPT, was 2.44 (SE = 0.16, *p* < 0.01). In this model, the effect of the CSI was negative and significant (beta = −0.07, SE = 0.00, *p* < 0.05). The effect of time was negative and not significant (beta = −0.03, SE = 0.02, *p* > 0.05). The effect of regionT was negative and significant (beta = −0.86, SE = 0.04, *p* < 0.01). The effects of the CSI, time, and region are visualized in Figure 1.

In the figure, the effects of time, CSI, and region are plotted to provide a summative overview. This indicates that, with lower CSI scores, the delta pain pressure thresholds increased after exercise, more visually in the trapezius region than the quadriceps. Furthermore, the pain pressure threshold scores were higher with lower CSI scores, as also indicated by the results. These interaction effects were also significant in the analyses. With higher CSI scores, the delta pain pressure thresholds presented to a lower pain pressure threshold score after exercise, eventually leading to a negative score. The interpretation was that, with lower CSI scores, exercise-induced hypoalgesia occurred, and with higher CSI scores, there was ineffective exercise-induced hypoalgesia. This effect was less visual in the quadriceps region, but was still present. Whether a change in the pain pressure threshold outcome was higher with lower CSI scores was, however, only suggested in limited amounts, and the strength of this conclusion was limited. Following the results of the CSI symptom severity calculator, only 11.8% of the sample were present in the classifications of (i) a low level of central sensitization-related symptom severity and (ii) medium level of central sensitization-related symptom severity. The rest of the sample (88.2%) was measured as having a high level of central sensitization-related symptom severity.

The model’s intercept, corresponding to the temporal summation, was 1.33 (SE = 0.54, *p* < 0.05). In this model, the effect of the CSI was positive and not significant (beta = −0.00, SE = 0.01, *p* > 0.05). The effect of time was positive and not significant (beta = 0.16, SE = 0.40, *p* > 0.05). When performing the variable selection based on the Akaike information criterion, every possible predictor was removed, resulting in a model with only an intercept. If the interaction between the CSI and time was kept in the model, the following conclusions were formed. There was an interaction effect between the CSI score and exercise-induced hypoalgesia measured with the delta pain pressure threshold at both the regions of the quadriceps and trapezius. However, the effect was small and insignificant, and more pronounced for the exercise-induced hypoalgesia measured in the trapezius region than the quadriceps. The results of the interaction effect show a non-significant negative effect (beta = −0.002) of the CSI on exercise-induced hypoalgesia measured with the delta pain pressure threshold, *t* (356) = −1.918, *p* > 0.05. There was no correlation between the CSI and exercise-induced hypoalgesia measured with temporal summations in either region. The results show a non-significant negative effect (beta = −0.004) of the CSI on exercise-induced hypoalgesia measured with the temporal summation, *t* (358) = −0.596, *p* > 0.05.

## 4. Discussion

This study aimed to improve our understanding of exercise-induced hypoalgesia in chronic WADs by examining the difference in the extent of exercise-induced hypoalgesia when subgroups are formed based on the CSI. We expected that the extent of exercise-induced hypoalgesia would be lower when more central sensitization symptoms were present. However, the analyses did not reveal the expected effects of exercise-induced hypoalgesia and the CSI. This was the case for both the pain pressure threshold and temporal summation measures. The analysis also showed no significant effect of time, meaning that there was no evidence for the presence of exercise-induced hypoalgesia on a group level in our population. Regarding the pain pressure threshold, the CSI related to the pain pressure threshold score. The temporal summation scores did not show any correlation.

Although we expected different results, it is well known that, in chronic pain populations, the pain response to exercise is variable. Conditioned pain modulation is believed to play an important role in the development and exacerbation of chronic pain because of the association with a shift in the balance between pain facilitation and pain inhibition. In patients with central sensitization, conditioned pain modulation is less efficacious, but also in healthy people it is highly variable [87]. Previous studies found suggestions of impaired exercise-induced hypoalgesia in individuals with WADs [23,26]. Related to that research, this study’s results align with these earlier findings. An additional aspect of this study was the influence of the CSI score on the presence of exercise-induced hypoalgesia. The findings suggest that central sensitization can contribute to impaired pain modulation. However, it is not clear what the correlation between central sensitization and exercise-induced hypoalgesia is in chronic WAD [88]. As it is known that exercise is an important part of rehabilitation in patients with chronic pain, the knowledge about the exact parameters of exercise is crucial. Nevertheless, there are insufficient insights into the appropriate types and levels of exercise for individual patients [8,74,89]. Therefore, we investigated the connection between the symptoms of central sensitization measured with the CSI and the difference in the occurrence of exercise-induced hypoalgesia. We expected that, in participants with higher CSI scores, exercise-induced hypoalgesia would be more disrupted. The results show the CSI’s effect on the delta pain pressure thresholds, suggesting that exercise-induced hypoalgesia is more present in patients with lower CSI scores and is more pronounced in the trapezius region. No correlations were found for temporal summation. However, the study had a limitation regarding the time predictor effect plot. Intending to investigate the scope of exercise-induced hypoalgesia in the subgroups, we divided the participants based on the CSI scores in our analyses. By doing so, we followed the advice from the authors who published the CSI symptom severity calculator [34]. In our sample, 88.2% were in the severe subgroup, 8.7% in moderate, and 3.1% in mild [34]. So, the analyses in our study did not have equal numbers of participants in the different subgroups, and the results of our research must be seen from that perspective. Given the mean duration of complaints since whiplash injury of the participants was 37.2 months in our sample, and the expectation that central sensitization is an aspect in the development and persistence of chronic pain, this was not unexpected [90,91]. We conducted this study in a rehabilitation center where patients with chronic complaints were treated, so the results applied to that group.

We can ask whether the CSI is an appropriate method to assess the correlation between impaired pain modulation to a patient’s response to exercise. Recently, the CSI was deemed a useless instrument for detecting deficits of conditioned pain modulation in patients with musculoskeletal pain due to the absence of a correlation with the psychophysical test results and the insufficient measurement of diagnostic accuracy [92]. This was consistent with our previous study investigating the validity of the CSI, which showed that the CSI was better equipped to identify the psychosocial factors related to central sensitization than the changes in the central nervous system [93]. This can mean that there may be a relationship between central sensitization and exercise-induced hypoalgesia, but the current method with the CSI is not sufficiently capable of identifying this relationship. We applied a test protocol for quantitative sensory testing in which the definite pain pressure threshold was the mean of two consecutive measures, following previous studies [64,70,94,95]. We also opted for this number of measurements to increase the feasibility of our study by limiting the burden on the participants. However, other research administers three measurements for pain pressure thresholds [96,97]. In retrospect, our decision was something to reconsider, as there was a weakness of performing two measurements, since the participants did not have the opportunity to become familiar with the measurements. The test protocol in this study included comprehensive information about the pain pressure threshold measurements. In future research, a test protocol with three pain pressure threshold measurements is preferable.

Studies investigating the effect of exercise-induced hypoalgesia use substantial heterogenic methodology [88]. In this study, we used the aerobic power index test, which was used in several studies to examine exercise-induced hypoalgesia in chronic pain populations, including chronic WAD [24,25,75]. However, it is unclear if the aerobic power index is a sufficient stimulus to trigger the mechanisms responsible for exercise-induced hypoalgesia. The studies mentioned stimuli durations varying in minutes, with different percentages of intensity. It was concluded that larger effect sizes for exercise-induced hypoalgesia were observed with longer duration of exercise (e.g., >10 min) in healthy individuals [21,98]. The results of this study could have possibly been different with a greater aerobic stimulus, because the participants hypothetically did not exercise enough to induce exercise-induced hypoalgesia. However, the intensity of the aerobic exercise in this study was considered quite stressful for most participants in the population measured. Longer durations of aerobic exercise were probably not tolerated, and we were convinced that we managed to create the stressful stimuli we wanted to achieve in this way. Nevertheless, because there is no gold standard, this choice might have influenced our results [99]. Smith and colleagues found that exercise-induced hypoalgesia occurred in people with chronic WADs in response to isometric exercise and not aerobic exercise [23]. This was consistent with other studies where isometric exercise resulted in reduced pressure sensitivity for shoulder pain [100]. Another study, however, determined exercise-induced hypoalgesia with aerobic exercise being performed for 4 min in people with chronic WADs [24]. Another aspect that could have influenced exercise-induced hypoalgesia was that the pain intensity in this study was higher than that reported in other comparable studies [23,26]. It has been suggested that patients reporting greater pain and disabilities may have less effective pain inhibition, however with a low body of evidence [26]. We can observe that our sample scores at the baseline are high for pain and disability. It could be that the occurrence of exercise-induced hypoalgesia was not present. So, a correlation would also be absent.

The participants demonstrated a balanced distribution of gender and age. Also, the sample was comparable to the outcomes of health-related quality of life [79,80,81,82,83] and neck pain disability [68,84,85] for other studies. The scores for cognitive functioning were lower than other studies on chronic WAD [50,68]. So, our sample seemed comparable to other cohorts in the research of people with chronic WAD.

The study’s fundings do not fully support our initial hypothesis, but the results offer insights into the nuanced relationship of central sensitization, exercise-induced hypoalgesia, and chronic WAD. The plotted figure of the interaction effects of time, region, and CSI provides some evidence for CSI’s influence on the delta pain pressure threshold. This observation can be interpreted as subtleties that help us to understand the complexity of conditioned pain modulation better. A limitation of this result was that the number of participants with a low score on the CSI was too small to achieve adequate conclusions on this topic. Further research on this subgroup is recommended, exploring the potential influence of the CSI score on exercise-induced hypoalgesia in people with chronic WADs and a low to medium level of central sensitization-related symptom severity. This can help us to achieve a better understanding of the usability in clinical practice.

## 5. Conclusions

Based on the results of this study, no interaction effect was observed between the CSI score and the occurrence of exercise-induced hypoalgesia. The plotted figure of the interaction effects of time, trapezius region, and CSI, though limited, provides some evidence for the influence of the CSI on the delta pain pressure threshold, but not for temporal summation purposes.

## 6. Strengths and Limitations

A notable limitation was that the majority of the sample exhibited high scores on the CSI. Consequently, the findings are especially meaningful for the subgroup experiencing severe central sensitization-related symptoms.

## 7. Recommendations

A suggested recommendation for further research involves replicating this study for a chronic WAD cohort with lower CSI scores, falling within the mild and moderate subgroups according to the CSI calculator of Cuesta-Vargas and colleagues [34]. It can be discussed to use another stimulus for quantitative sensory testing measurements.

## Figures and Tables

**Table 1:** Demographic characteristics (*n* = 135).

Age (M [SD]) (years)	38.7 [11.1]
Sex, no (%)	
Women (%)	77 (57.0)
Men (%)	58 (43.0)
Time since whiplash injury (*M* [SD]) (months)	37.20 [11.1]
Type of whiplash injury, no (%)	
Motor vehicle accident	126 (93.3)
Bicycle accident	3 (2.2)
Scooter accident	1 (0.7)
Collision with an animal (dog)	1 (0.7)
Bumped into a garage door	1 (0.7)
Physical violence; kicked	2 (1.5)

n—number of participants, M—mean, SD—standard deviation, no—number.

**Table 2:** Physical characteristics of the participants.

	Scale Range	N	Mean Score (SD)
RAND36_general health_	0–100	120	50.71 (21.13)
RAND36_health change_	0–100	120	22.71 (29.53)
RAND36_physical functioning_	0–100	120	53.42 (22.20)
RAND36_role physical_	0–100	120	10.00 (21.35)
RAND36_role emotional_	0–100	120	46.91 (38.59)
RAND36_social functioning_	0–100	120	39.19 (26.07)
RAND36_pain_	0–100	120	29.23 (17.44)
RAND36_vitality_	0–100	120	35.17 (18.63)
RAND36_mental health_	0–100	120	56.13 (20.22)
NDI	0–50	127	26.55 (7.54)
mPDQ	0–72	117	51.42 (16.56)
CSI	0–100	127	52.61 (13.99)
VAS	0–100	123	65.98 (20.08)

N—number of participants from whom data was collected, RAND36—RAND 36-item Health Survey, NDI—neck disability index, mPDQ—modified perceived deficits questionnaire, CSI—central sensitization inventory, VAS—visual analog scale.

**Table 3:** QST measurements before and after aerobic exercises.

Measurement	N	Time Point	Mean (SD)	Range	
				Min.	Max.
PPT region trapezius, kg/cm^2^	132	pre-exercise	2.62 (1.82)	0.42	10.91
	128	post-exercise	2.91 (2.50)	0.17	20.80
PPT region quadriceps, kg/cm^2^	132	pre-exercise	7.98 (5.01)	0.89	21.97
	128	post-exercise	8.07 (5.71)	0.30	30.10
TS region trapezius, VNRS	131	pre-exercise	1.43 (1.70)	−3	6
	128	post-exercise	1.41 (1.74)	−5	6
TS region quadriceps, VNRS	131	pre-exercise	1.66 (1.90)	−3	9
	128	post-exercise	1.59 (1.82)	−3	6

N—number of participants from whom data was collected, PPT—pressure pain threshold, TS—temporal summation, SD—standard deviation, min.—minimum score, max.—maximum score, VNRS—verbal numeric rating scale.

## Data Availability

Data are unavailable due to privacy restrictions.
